# Chloroplastic photoprotective strategies differ between bundle sheath and mesophyll cells in maize (*Zea mays* L.) Under drought

**DOI:** 10.3389/fpls.2022.885781

**Published:** 2022-07-14

**Authors:** Wen-Juan Liu, Hao Liu, Yang-Er Chen, Yan Yin, Zhong-Wei Zhang, Jun Song, Li-Juan Chang, Fu-Li Zhang, Dong Wang, Xiao-Hang Dai, Chao Wei, Mei Xiong, Shu Yuan, Jun Zhao

**Affiliations:** ^1^Institute of Quality Standard and Testing Technology Research, Sichuan Academy of Agricultural Sciences, Chengdu, China; ^2^Biotechnology Research Institute, Chinese Academy of Agricultural Sciences, Beijing, China; ^3^College of Life Sciences, Sichuan Agricultural University, Ya’an, China; ^4^Plant Science Facility of the Institute of Botany, Chinese Academy of Sciences, Beijing, China; ^5^College of Resources Science and Technology, Sichuan Agricultural University, Chengdu, China

**Keywords:** bundle sheath chloroplast, drought stress, maize (*Zea mays* L.), non-photochemical quenching, photoprotection, reactive oxygen species

## Abstract

Bundle sheath cells play a crucial role in photosynthesis in C4 plants, but the structure and function of photosystem II (PSII) in these cells is still controversial. Photoprotective roles of bundle sheath chloroplasts at the occurrence of environmental stresses have not been investigated so far. Non-photochemical quenching (NPQ) of chlorophyll a fluorescence is the photoprotective mechanism that responds to a changing energy balance in chloroplasts. In the present study, we found a much higher NPQ in bundle sheath chloroplasts than in mesophyll chloroplasts under a drought stress. This change was accompanied by a more rapid dephosphorylation of light-harvesting complex II (LHCII) subunits and a greater increase in PSII subunit S (PsbS) protein abundance than in mesophyll cell chloroplasts. Histochemical staining of reactive oxygen species (ROS) suggested that the high NPQ may be one of the main reasons for the lower accumulation of ROS in bundle sheath chloroplasts. This may maintain the stable functioning of bundle sheath cells under drought condition. These results indicate that the superior capacity for dissipation of excitation energy in bundle sheath chloroplasts may be an environmental adaptation unique to C4 plants.

## Introduction

Plant growth and productivity are adversely affected by various abiotic and biotic stress factors in both natural and agricultural ecosystems. Photosynthesis is the primary physiological process that drives plant growth and crop productivity and influences many other plant processes. Studies have indicated that the photosynthetic apparatus of higher plants is highly susceptible to environmental stresses such as high light intensity, cold, UV radiation, high salinity, and water deficit. Soil drought is an important limitation that severely impairs plant growth, development, crop yield, and various morphological, anatomical, physiological, and biochemical processes. Inhibition of photosynthesis is one of the primary physiological consequences of drought stress. Reports show that during water deficit, plants experience a number of metabolic changes that affect photosynthesis, including stomatal closure (Campos et al., 2014), decline in the content of photosynthetic pigments (Hsu et al., 2003), production of reactive oxygen species (ROS; Chen et al., 2016), and limitation of photosynthetic carbon metabolism (Dias and Brüggemann, 2010).

The photosynthetic apparatus of higher plants comprises two chlorophyll-protein complexes photosystem I (PSI) and photosystem II (PSII), which are located in the thylakoid membranes. PSII catalyzes the light-driven electron transfer from water to plastoquinone. When the energetic balance of chloroplasts changes, there are two major mechanisms in PSII to sense and respond. One is the strong and reversible phosphorylation of several proteins in the PSII-light-harvesting complex II (LHCII) supercomplexes, and the other is non-photochemical quenching (NPQ) of chlorophyll a (Chl a) fluorescence (Tikkanen and Aro, 2012). Together, these regulatory mechanisms maintain the energetic balance of the electron transfer reactions, prevent excess energy from damaging photosynthetic apparatus, and lead to the migration and reorganization of the PSII-LHCII complexes along the thylakoid membrane. It has been demonstrated that PSII can dissipate excess absorbed light energy into heat through enhancing NPQ in response to water deficit (Liu et al., 2009; Chen et al., 2016). However, the regulatory mechanism of NPQ *in vivo* under drought stress remains to be elucidated. The reversible phosphorylation and metabolism of PSII functional proteins in *Arabidopsis thaliana* and barley (*Hordeum vulgare* L.) cultivars under water stress were discovered in our previous research. The repair cycle process of damaged reaction centers of PSII (RCII) under water stress appeared to be different from that under high-light treatment (Yuan et al., 2005; Liu et al., 2009; Chen et al., 2016). Furthermore, in nature, drought conditions are frequently accompanied by other environmental stressors such as irradiation, elevated temperature, and nutrient deficiency, which can result in more complicated photoprotection responses of the photosynthetic apparatus.

Some pathways that regulate the responses of photosynthesis to environmental stress have been established in C3 plants such as Arabidopsis, beans, and cereal crops (wheat, barley, and rice). C4 plants have two distinct chloroplast types, mesophyll and bundle sheath chloroplasts, which cooperate to accomplish photosynthesis. There has been controversy surrounding the structure and function of PSII in the bundle sheath chloroplasts of C4 plants. Some earlier reports considered that bundle sheath chloroplasts of C4 plants lack grana and display depleted PSII activity owing to the absence of polypeptides participating in the water oxidation or light harvesting of PSII (Hatch, 1987). However, some opposing reports have revealed that the bundle sheath chloroplasts of some C4 plants did contain a significant capacity for O_2_ evolution and NADP^+^ reduction linked with PSII (Chapman et al., 1980). Additionally, the excitation energy of PSII has been shown to be efficiently transferred to PSI in the bundle sheath thylakoids of many C4 plants (Pfündel et al., 1996). Maize is a typical C4 plant and is one of the most cultivated crops worldwide. In 2006, Romanowska et al. effectively isolated the mesophyll and bundle sheath chloroplasts of maize. They then revealed that PSII in the bundle sheath thylakoids contained all the polypeptides involved in photosynthetic electron transport and oxygen evolution, albeit the abundance and activity of the PSII complex were very low. Moreover, the reversible phosphorylation of PSII-LHCII proteins and the degradation of damaged D1 proteins were observed in isolated mesophyll and bundle sheath chloroplasts of maize under high light conditions (Pokorska et al., 2009). This demonstrated that the repair cycle of RCII could exist in the two cell types.

In this paper, we report on the NPQ mechanism of PSII in the bundle sheath chloroplasts of maize. Additionally, we demonstrate the superior capacity for excess energy dissipation of bundle sheath chloroplasts compared to that of mesophyll chloroplasts, under progressive drought stress. In both mesophyll and bundle sheath chloroplasts, the NPQ increased with the intensity of the drought treatment. This was accompanied by the dephosphorylation of LHCII and an increase in PSII subunit S (PsbS) content. The comparison of the two types of chloroplasts showed that under drought conditions, there was less accumulation of ROS in bundle sheath cells, and the PSII complexes and chloroplast structures were more stable. The photoprotection in bundle sheath chloroplasts may be beneficial for maintaining the photosynthetic efficiency and the capacity for transporting nutrients in maize leaves subjected to drought stress.

## Materials and methods

### Plant materials and stress treatments

Maize (*Zea mays* L.) inbred line Zheng 58 was used in this work. Maize plants were potted in a growth chamber under a 14 h photoperiod, with relative humidity 70%, an irradiance of 300 μmol photons m^−2^ s^−1^, and a day/night regime of 24/22°C.Maize seeds were surface sterilized in 1% sodium hypochlorite for 10 min. Before sowing, the seeds were imbibed and incubated on moisture filter paper at 24°C in dark for 48 h, then were sown into pots (60 cm × 20 cm × 16 cm) filled with compost soil. Ten maize plants were grown in each pot. Loamy soil (pH 6.2–6.9) was used in this study. Soil organic matter, alkali-hydrolyzed nitrogen, available phosphorus, and available potassium were 18.7 g kg^−1^, 157 mg kg^−1^, 9.7 mg kg^−1^, and 177 mg kg^−1^, respectively. When grown to the fourth-leaf stage, drought stress was imposed on maize plants through withholding water. The experiment consisted of four soil moisture regimes with four replications. (i) Well-watered control (CK): pots were irrigated sufficiently to maintain soil water content at 80%–85% of field water capacity (FWC). (ii) Mild drought stress (S1): water content in the entire soil profile was maintained at 70%–75% FWC. (iii) Moderate drought stress (S2): water content in the entire soil profile was maintained at 50%–55% FWC. (iv) Severe drought stress (S3): water content in the entire soil profile was maintained at 30%–35% FWC. To measure the FWC, experimental soil was taken by ring knife and subjected to water absorption water for 24 h. The soil of saturated water absorption was weighted (w1), and dried at 105°C for more than 10 h, after which, dry soil was determined (w2). Field water capacity was calculated as: FWC = (w1-w2) w2^−1^ × 100%. The FWC of the soil in the experiment was 87.83%. During withholding irrigation, the soil relative water content in each pot was measured every 3 days till the individual moisture regimes were achieved. Then, the soil moisture was maintained by measuring gravimetrically every day. After 7 days of drought stress, the first developed leaf at the top of maize plant was harvested for following experiments.

### Leaf water status and chlorophyll contents

The results of stress to plants were characterized by the relative water content (RWC) of leaves. RWC was measured according to the method of Tambussi et al. (2005) with minor modifications. Leaves of maize were weighed (wi), floated on distilled water at 4°C overnight, weighed again (wf), and dried at 80°C–90°C for 4–6 h, after which, dry mass was determined (wd). Relative water content was calculated as: RWC = (wi-wd) (wf-wd)^−1^ × 100%.

Chlorophyll (Chl) a and b content was measured according to the method of Lichtenthaler (1987) with some modifications. Fifteen fresh leaf disks (1 cm^2^ each) were taken with hole punch from each sample leaf, then were cut into filaments (5 mm × 1 mm) and put into tubes with 5 ml of 80% acetone. The leaf and acetone were incubated at room temperature for 24 h in darkness to allow for complete extraction of chlorophyll into the solution. The absorbance of the extract was measured in microtiter plate using a microplate reader (Synergy H1, BioTek, Vermont, United States) at 645 and 663 nm. Calculate the concentration of Chl a and Chl b in the cuvette using the equations: Chl a (μg ml^−1^) = 12.7 *A*_663_ −2.69 *A*_645_, Chl b (μg ml^−1^) = 22.9 *A*_645_ −4.68 *A*_663_. The total chlorophyll content based on leaf area in original suspension was calculated as: Chl (μg cm^−2^) = (Chl a + Chl b) × 5/S, where S (cm^−2^) was the value of leaf area.

### Assay of reactive oxygen species

Superoxide anion radicals (O_2_^−^) and hydrogen peroxide (H_2_O_2_) levels were visually detected with nitro-blue tetrazolium (NBT) and 3,3-diaminobenzidine (DAB), respectively, as described in Chen et al. (2016) with some modifications. Leaf tissues were excised into segments (8 cm) without the tip and base parts and then immersed into 6 mM NBT solution containing 50 mM Hepes buffer (pH 7.5) for 2 h or 1 mg ml^−1^ DAB solution (pH 3.8) 1 day in the dark. Leaves stained by NBT or DAB solution were decolorized in boiling ethanol (80%) or 70°C ethanol (95%) respectively for chlorophyll removal. Finally, leaves-segments stained by NBT and DAB solution were crosscut with a razor blade (avoiding the thick midrib), and the transection sections were observed and imaged through the microscope (Biological microscope L1800, Guangzhou LISS Optical Instrument Co., Ltd., Guangzhou, China). The microscopic images in mesophyll and bundle sheath cells after staining with NBT and DAB were quantitative analyzed using the Image J software. More than five “Kranz structure” cell regions were selected from leaf transection to analyze the depth of staining per unit area.

The production of O_2_^−^ was determined using the method of Elstner and Heupel (1976) by monitoring the nitrite formation from hydroxyl amine. The content of H_2_O_2_ was measured according to the method of Okuda et al. (1991). Approximately 0.5 g of fresh leaf tissues was cut into small pieces and homogenized in an ice bath with 5 ml of 0.1% (w/v) TCA. After centrifugation at 12,000 × *g* for 20 min at 4°C, 0.5 ml of supernatant plant extract was added to 0.5 ml of 10 mM potassium phosphate buffer (pH 7.0) and 1 ml of 1 M KI. The absorbance of the supernatant was recorded at 390 nm. Finally, the concentration of H_2_O_2_ was calculated using a standard curve plotted with known concentrations of H_2_O_2_.

### Lipid peroxidation measurements

The level of lipid peroxidation in maize leaves from each treatment was estimated by measuring electrolyte leakage and malondialdehyde (MDA) contents. To determine electrolyte leakage, 0.5 g fresh leaf samples were cut into 5–10 mm length and put in test tubes containing 25 ml deionized water. The tubes were covered with caps and placed in a vacuum-pumping equipment for 20 min, to measure the initial electrical conductivity (EC1) of leaves using an electrical conductivity meter (DDS-307, RuiZi, Chengdu, China). The samples were heated at 100°C for 30 min to completely kill the tissues and release all electrolytes. After cooling to room temperature, the final electrical conductivity (EC2) of leaves was measured. Electrolyte leakage (EL) was expressed following the formula EL = EC1/EC2 × 100%.

The thiobarbituric acid (TBA) method was applied to measure MDA concentration in leaf cells or in mesophyll and bundle sheath chloroplasts. Fresh leaf tissues (1.0 g) were homogenized in 10 ml of 10% (w/v) trichloroacetic acid (TCA). The homogenate was centrifuged at 4°C for 10 min at 4,000 r min^−1^. To 2 ml of the supernatant, 2 ml of 0.6% TBA was added. The assay mixture was heated at 95°C for 15 min and then quickly cooled in an ice bath. The mixture was centrifuged at 4,000 r min^−1^ for 15 min at 4°C. The concentration of leaf total MDA was calculated from the difference of the absorbance of the supernatant at 532 and 600 nm. For measuring plastid MDA, mesophyll and bundle sheath chloroplasts of maize leaves were isolated mechanically according to Romanowska and Parys (2011). Before homogenization in 10% TCA, the Chl concentrations of two chloroplasts preparations were measured in 80% (v/v) acetone.

### Gas exchange

Gas exchange analysis of maize leaves was made using an open system (CIRAS-3, PP system, Hitchin, United Kingdom) from 9 am to 12 am each day for 3 days. All measurements were taken at a constant airflow rate of 300 ml min^−1^ under the artificial light condition of 1,200 μmol m^−2^ s^−1^. The reference concentration of ambient CO_2_ was about 400 μmol mol^−1^, the temperature was 25°C, and the relative humidity was 30%. Photosynthetic parameters including stomatal conductance, transpiration rate, intercellular CO_2_ concentration, and net photosynthetic rate were measured.

### *In vivo* chlorophyll fluorescence measurements

Chlorophyll a fluorescence induction kinetics and imaging of intact leaves were measured at room temperature with a pulse-amplitude-modulated imaging fluorometer (the Imaging PAM M-Series Chlorophyll Fluorescence system, Heinz Walz GmbH, Effeltrich, Germany). Maize plants were dark adapted for 30 min prior to fluorescence measurements. The minimal fluorescence yield (Fo) and maximal fluorescence yield (Fm) were measured with dark-adapted leaves when all RCII are fully opened and closed. Fm was induced by a saturating pulse of white light (0.8 s, 8,000 μmol m^−2^ s^−1^). The fluorescence in stable state (Fs) was measured after actinic light (1,000 μmol m^−2^ s^−1^) was applied for 30 min. Then the maximal fluorescence yield after light adaption (Fm’) was attained with a pulse of saturating light with 0.8 s interval while leaves were illuminated by actinic light. After the actinic light was turned off, a far-red light was applied to excite PSI preferentially, leaving the electron transport of PSII in an oxidized state, to obtain the minimum fluorescence of the light-adaptive leaves (Fo’). By using fluorescence parameters determined in both light- and dark-adapted leaves, the maximal photochemical quantum yield of PSII in darkness [Fv/Fm = (Fm-Fo)/Fm], the maximal photochemical quantum yield of PSII under light [Fv’/Fm’ = (Fm’-Fo’)/Fm’], the effective photochemical quantum yield of PSII under light [Φ_PSII_ = (Fm’-Fs)/Fm’], and the non-photochemical fluorescence quenching under light [NPQ = (Fm-Fm’)/Fm’] were calculated. Among these parameters, Fv/Fm is used to monitor the potential efficiency of PSII photochemistry, Φ_PSII_ represents light use efficiency at a given light intensity, and NPQ reflects heat-dissipation of excitation energy in the antenna system. The image data averaged in each experiment were normalized to a false color scale. Light responses curves (LRCs) analysis was performed using light steps between 0 and 1,500 mol m^−2^ s^−1^. During light-to-dark shifts, NPQ kinetics of intact maize leaves was measured according to Chen et al. (2019).

### Chlorophyll fluorescence kinetic microscope measurements

In maize, a typical C4 plant, it was demonstrated that bundle sheath chloroplast contained about half the amount of PSII complexes compared with mesophyll one (Romanowska et al., 2006). Nevertheless, it is impossible to distinguish chlorophyll fluorescence of bundle sheath chloroplasts from mesophyll chloroplasts, on the intact leaves, due to leaf anatomy in which a few layers of mesophyll cells tightly surround one layer of bundle sheath cells. In this study, the chlorophyll fluorescence kinetic microscope (FKM) system (Micro-FluorCam FC2000, Photon Systems Instruments, Brno, Czech Republic) was applied to analyze the fluorescence parameters at the cellular level *in vivo*.

Fresh leaves with different treatments were carefully crosscut, and the transection of slices (more than three slices of each sample) were imaged and measured under microscope. The FKM was operated by the FluorCam software, and the modules of slow kinetics, light responses curves, and light-to-dark shifts were applied. Blue excitation (470 nm) was used and Chlorophyll fluorescence was detected from 695 to 770 nm. Measuring saturating and actinic light were 3,000 and 180 μmol m^−2^ s^−1^, respectively. From the microscopic imaging of the leaf transection, different types of cell regions were selected to individually analyze the fluorescence parameters of mesophyll or bundle sheath chloroplasts, in which more than five “Kranz structure” cell regions were selected from each slice. Consistent with the experiment of intact leaves, parameters such as Fo, Fm, Fo’, Fm’, and Fs were measured. Fv/Fm, Fv’/Fm’, Φ_PSII_ and NPQ of mesophyll and bundle sheath chloroplasts were calculated.

### Electron microscopy

Leaves of maize plants with differently drought treatments were cut into pieces about 2 mm × 3 mm, and three of which were fixed immediately with 3% glutaraldehyde in 0.1 M sodium cacodylate buffer (pH 6.9) at 4°C over night. The fixed leaves pieces were then post-fixed with 1% osmium tetroxide, dehydrated in series acetone and embedded in Epon 812, as described previously (Liu et al., 2009). Ultrathin sections, cut with an ultramicrotome (EM UC6, Leica Microsystem GmbH, Wetzlar, Germany), were observed with a transmission electron microscope (H-7500, Hitachi, Tokyo, Japan) operating at 75 kV. Three visual field of each section were selected to observe.

### Thylakoid isolation

Mesophyll and bundle sheath thylakoids were isolated according to Romanowska and Parys (2011). Isolation procedures were carried out at 4°C, under dim green light. All the isolation buffers were supplemented with 10 mM NaF to inhibit thylakoid protein dephosphorylation. Maize leaves were homogenized in a medium containing 400 mM mannitol, 50 mM HEPES-NaOH (pH 8.0), 5 mM MgCl_2_, 10 mM NaCl, 10 mM sodium isoascorbate, 2 mM PMSF, 5 mM amino-n-caproic acid (EACA), 1 mM benzamidine (BA), 2 mM EDTA, 10 mM NaF, and 0.2% (w/v) BSA. The homogenate was filtered through six layers of Miracloth (20 mm) and the filtrate was used for preparation of mesophyll chloroplasts. The residue continued to be homogenized, then filtered and the residue on the cloth was washed briefly with cold distilled water until the filtrate was clear. The residue was microscopically examined to ensure that the bundle sheath strands were completely free of mesophyll contamination. The final bundle sheath residue was homogenized and filtered through six layers of nylon (20 mm). The filtrates obtained during isolation of mesophyll chloroplasts and bundle sheath chloroplasts were centrifuged at 8,000 × *g* for 15 min. Isolated thylakoid samples were immediately frozen in liquid nitrogen and stored at −80°C until use. The effective isolation of mesophyll and bundle sheath cells was monitored by immuno detection of anti-phosphoenolpyruvate carboxylase (PEPC) and anti-Ribulose-1.5-bisphosphate carboxylase/oxygenase (Rubisco), which are key enzymes of CO_2_ assimilation process located in mesophyll and bundle sheath cells, respectively.

### Sds-page and immunoblot analysis

According to the method of Pokorska et al. (2009) and Betterle et al. (2015) with minor modification, isolated thylakoid samples were solubilized in the denaturing buffer containing 0.05 M Tris–HCl (pH 6.8), 5% (w/v) SDS, 8 M urea, 5% (v/v) 2-mercaptoethanol, and 20% (v/v) glycerol. The polypeptides were separated by SDS-PAGE using 15% (w/v) acrylamide gels with 3 or 6 M urea. The amount of protein loaded was equivalent to 0.5–1.5 μg of Chl depending on the protein abundance in thylakoid membranes. For western blotting, separated proteins were electro-transferred onto a PVDF membrane (Immobilon, Millipore, Massachusetts, United States). Then antibodies against D1, D2, CP43, CP47, Lhcb1, Lhcb2, Lhcb4, PsbS (Agrisera, Vännas, Sweden), and a polyclonal anti-phosphothreonine antibody (Cell Signaling Technology, Boston, United States) were applied. The signals were revealed by using a chemiluminescent detection system (ECL, GE Healthcare, Buckinghamshire, United Kingdom). Quantification of the immunoblots was done using Lane 1D analysis software (SAGE Creation, Beijing, China).

### *In vivo* dephosphorylation of thylakoid proteins

According to Rokka et al. (2000) and Liu et al. (2009), maize plants were illuminated under a PFD of 1,000 μmol photons m^−2^ s^−1^ at 25°C for 60 min to phosphorylate RCII proteins. To induce maximal LHCII phosphorylation, maize leaves were illuminated at low light (a PFD of 80 μmol photons m^−2^ s^−1^) for 60 min. Metal halide lamps were served as light source. After light treatment the maize leaves were transferred to darkness and incubated at 25°C for up to 120 min for gradual dephosphorylation. Samples for mesophyll and bundle sheath thylakoids isolation were taken during the time course of incubation, frozen in liquid nitrogen, and stored at −80°C.

### Blue native page

Thylakoid protein solubilization and BN-PAGE analysis was performed as described by Pokorska et al. (2009) with slight modification. Thylakoid membranes, corresponding to 15 μg Chl, were sedimented at 7,000 g for 5 min at 4°C and resuspended in 25 mM Bis Tris-HCl (pH 7.0), 20% (v/v) glycerol. Membrane proteins were solubilized by the addition of n-dodecyl β-D-maltoside (DDM) in 25 mM imidazole-HCl (pH 7.0), 20% glycerol, to a final concentration of 1% (w/v) for mesophyll thylakoid and 2% (w/v) for bundle sheath one. Final chlorophyll concentration was 0.5 mg/ml. Samples were incubated on ice for 10 min followed by centrifugation at 18,000 × *g* for 15 min. The supernatant was supplemented with 1/10 volume of BN sample buffer (5% w/v Serva Blue G, 100 mM Bis Tris-HCl pH 7.0, 30% w/v sucrose and 500 mM ε-amino-n-caproic acid) and loaded directly onto a 4%–12% acrylamide (w/v) gradient gel. Electrophoresis was performed at 4°C by increasing the voltage gradually from 50 to 200 V during the 3–4 h run. After the BN- PAGE run, the immunoblotting of thylakoid membrane proteins was performed according to the method of Wittig et al. (2006). The quantitative analysis of thylakoid membrane complexes was performed using Lane 1D software.

### Statistical analysis

At least four independent replicates were conducted for each determination. Data analysis was performed using the statistical software SPSS 17.0, and the means were compared using Duncan’s multiplication range test. A difference was considered to be statistically significant when *p* < 0.05. Error bars in figures represent SD of the means.

## Results

### Response of the water status, chlorophyll content, and gas exchange to drought stress in maize leaves

Drought stress was induced by withholding water according to four different soil moisture regimes. These included a well-watered control (CK), mild drought stress (S1), moderate drought stress (S2), and severe drought stress (S3). The relative water content (RWC) of the maize leaves decreased gradually with increasing drought stress (Supplementary Figure 1A). This indicated that the decrease in soil moisture led to the water deficit in maize leaves.

A decrease in chlorophyll content is commonly observed under drought stress (Yuan et al., 2005; Liu et al., 2009; Chen et al., 2016). As shown in Supplementary Figure 1B, mild drought stress did not have an obvious effect on the Chl a and b contents, but the total chlorophyll content was markedly reduced under moderate and severe drought conditions.

The stomatal conductance (*g_s_*), transpiration rate (*E*), intercellular CO_2_ concentration (*C_i_*), and CO_2_ assimilation rate (*A*) were measured in maize leaves under different drought conditions (Supplementary Figures 1C–F). Compared with the control, *g_s_* and *E* gradually declined under progressive drought stress. Mild or moderate drought stress did not have an obvious influence on *C_i_*, but *C_i_* increased significantly under severe drought stress. *A* displayed a slight decrease under mild drought stress; this was not statistically significant. However, moderate and severe drought stress dramatically reduced *A* in maize leaves.

### Drought stress induced significant ros accumulation and lipid peroxidation in maize mesophyll cells

**Figure 1:**
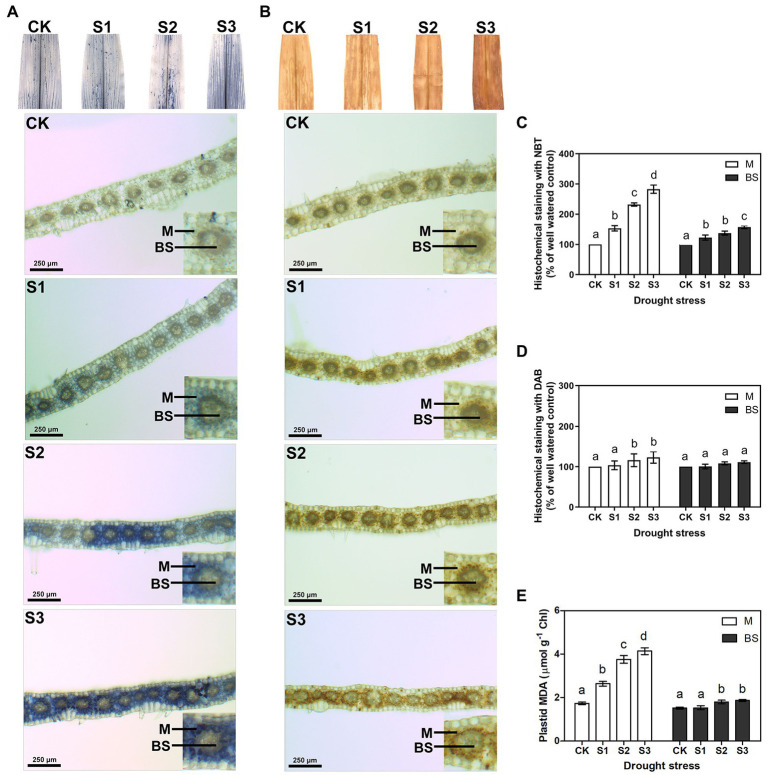
Measurement of reactive oxygen species (ROS) and lipid peroxidation in mesophyll (M) and bundle sheath (BS) cells of maize leaves under drought stress. Histochemical assays for O_2_^−^ and H_2_O_2_ in leaves by nitro-blue tetrazolium (NBT; **A**) and 3,3-diaminobenzidine (DAB; **B**) staining, respectively. Distribution of O_2_^−^ and H_2_O_2_ in M and BS cells were imaged in microscope. The microscopic images in M and BS cells were quantitative analyzed **(C,D)**. Plastid malondialdehyde (MDA) content (**E**) in M and BS chloroplasts of maize leaves were detected. CK, S1, S2, and S3 represent, respectively, the soil moisture regimes of well-watered, mild drought stress, moderate drought stress and severe drought stress. Values are expressed as the means ± SD from four independent biological replicates (*n* = 4). Different letters depict significant differences between the treatments (*p* < 0.05) according to Duncan’s multiplication range test.

The accumulation of ROS can be detected in plants under environmental stress (Foyer and Noctor, 2005; Rao and Chaitanya, 2016). To identify the influence of drought stress on ROS production, in this experiment, the levels of the two major ROS, O_2_^−^ and H_2_O_2_, were measured by quantitative analysis and histochemical staining of maize leaves. To further compare the differences in ROS accumulation between mesophyll and bundle sheath cells in maize leaves under drought stress, the transverse sections of stained leaves were observed under a light microscope. As shown in Supplementary Figures 2A,B, both O_2_^−^ and H_2_O_2_ levels increased when the maize plant was exposed to drought stress, particularly when exposed to severe drought stress. Interestingly, compared with bundle sheath cells, ROS accumulation in mesophyll cells of maize leaves increased more obviously, especially under moderate and severe drought conditions (Figures 1A–D).

ROS accumulation can damage membrane lipids. Hence, in this experiment, electrolyte leakage and malondialdehyde (MDA) contents were examined to estimate the amount of lipid peroxidation and to further determine the degree of oxidative damage on the plants. As shown in Supplementary Figures 2C,D, the electrolyte leakage and the total leaf MDA content increased significantly under moderate and severe drought stress. This indicated that ROS accumulation increased the lipid peroxidation in maize leaves under serious drought conditions. Notably, the MDA content in the mesophyll chloroplasts displayed a clear increase under drought stress, whereas the MDA content in bundle sheath chloroplasts was less affected by the drought treatments (Figure 1E).

### The npq in bundle sheath chloroplasts increased more markedly than in mesophyll chloroplasts under drought stress

**Figure 2:**
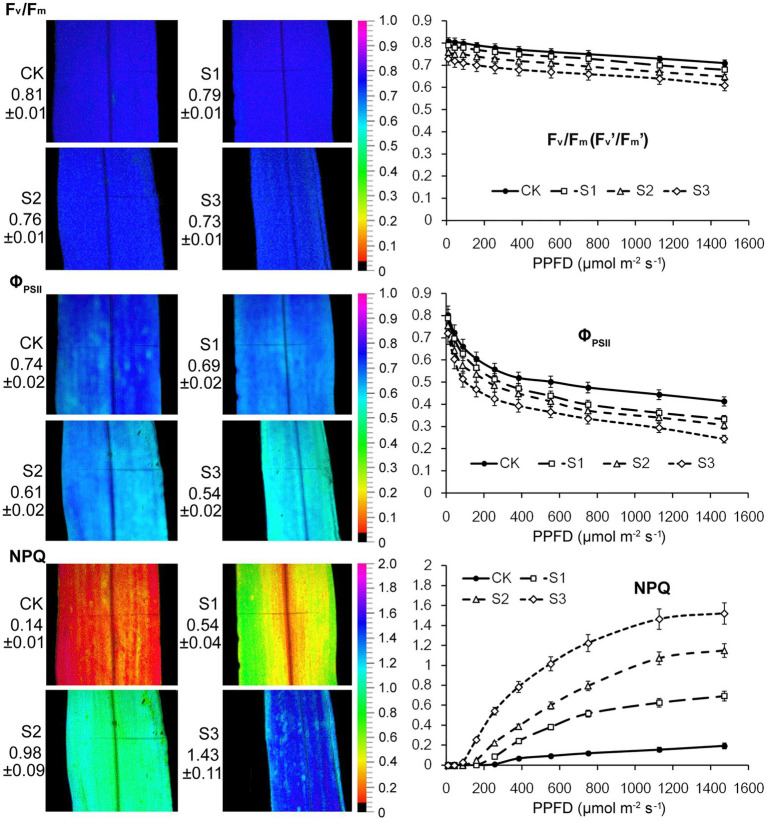
Chlorophyll fluorescence imaging and light response curves (LRCs) in intact maize leaves under drought stress. Fv/Fm, the maximal photochemical quantum yield of PSII in darkness; Fv’/Fm’, the maximal photochemical quantum yield of PSII under light; Φ_PSII_, the effective photochemical quantum yield of PSII under light; NPQ, the non-photochemical fluorescence quenching under light. CK, well-watered control; S1, mild drought stress; S2, moderate drought stress; and S3, severe drought stress. Values beside the individual images present quantitative means ± SD (*n* = 4). Vertical bars represent SD of the mean (*n* = 4).

The ratio obtained from chlorophyll fluorescence parameters, Fv/Fm, reflects the primary conversion efficiency of light energy in PSII. It is also an excellent indicator for measuring the degree of photoinhibition. As shown in Figure 2, there were no statistically significant changes in the Fv/Fm value when plants were subjected to mild drought stress. After severe drought, the Fv/Fm value decreased from 0.81 to 0.73. This resulted from the reduction in the proportion of open RCIIs under severe drought stress. The changes observed in Fv’/Fm’ and Φ_PSII_ of PSII further indicated that severe drought stress suppressed the electron transfer capability of PSII. Thermal energy dissipation from the antenna pigments of PSII, as an important mechanism that protects against excessive excitation, increased when plants were subjected to the drought stress.

**Figure 3:**
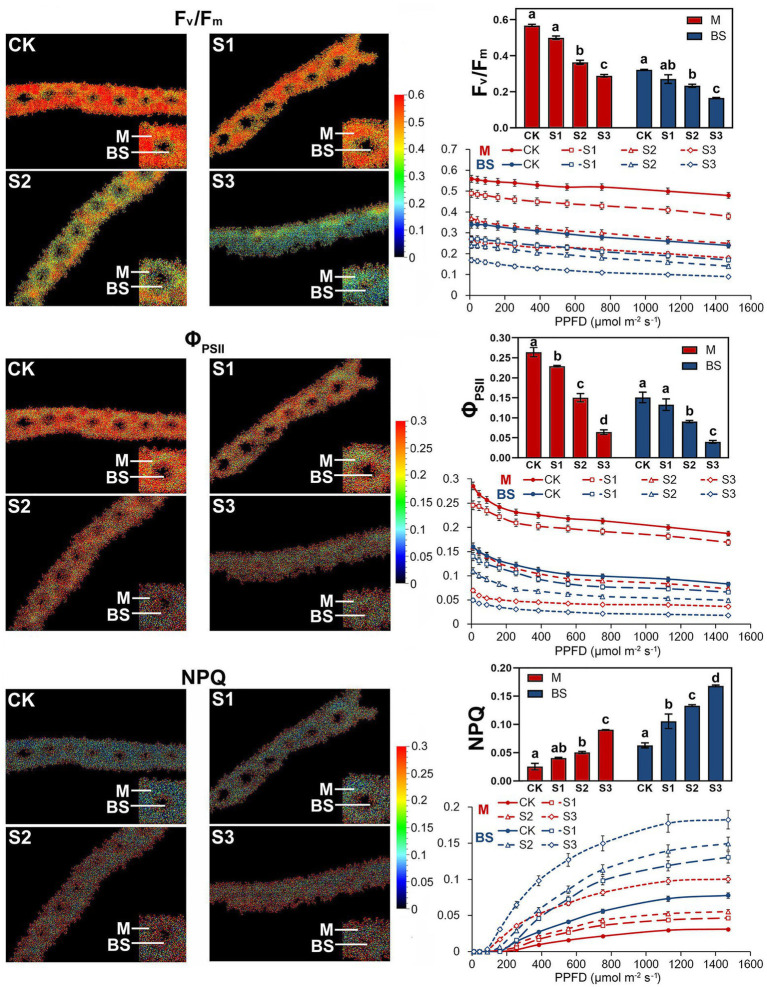
Chlorophyll fluorescence imaging and LRCs in M and BS chloroplasts of maize leaves under drought stress. Fv/Fm, the maximal photochemical quantum yield of PSII in darkness; Fv’/Fm’, the maximal photochemical quantum yield of PSII under light; Φ_PSII_, the effective photochemical quantum yield of PSII under light; NPQ, the non-photochemical fluorescence quenching under light. CK, well-watered control; S1, mild drought stress; S2, moderate drought stress; and S3, severe drought stress. Vertical bars represent SD of the mean (*n* = 4). Different letters mean significant differences at the 0.05 level according to Duncan’s multiplication range test.

Fluorescence Kinetic Microscope (FKM) measurements have been applied to analyze the *in vivo* photosynthetic activity of individual cells in algae (Ferimazova et al., 2013) and plants (Jacobs et al., 2016). In recent years, the application of FKM measurements to determine the Chl a fluorescence of plant mesophyll and bundle sheath cells has also been reported (Gorecka et al., 2014). In this study, Chl a fluorescence in the mesophyll and bundle sheath cells of maize leaves was detected by FKM measurement. Consistent with the results of previous studies (Ferimazova et al., 2013; Gorecka et al., 2014; Jacobs et al., 2016), at the microscopic level, the values of chlorophyll fluorescence, including Fv/Fm, ΦPSII and NPQ, were all much lower than those measured on intact leaves. The reasons for the low values of chlorophyll fluorescence, especially NPQ values, measured with FKM in this study may be the low chlorophyll contents per unit area in leaf slices, and the low actinic light intensity applied to avoid photoinhibition to leaf slices. As shown in Figure 3, under severe drought stress, the Kranz structure of the maize leaves was looser compared to that of the control plants. Mild drought stress did not significantly affect the photochemical efficiency of PSII in mesophyll or bundle sheath cells. However, the Fv/Fm and ΦPSII values in the two classes of chloroplasts declined markedly when plants were exposed to moderate and severe drought conditions. These results were consistent with those of the intact leaf (Figure 2). NPQ in mesophyll and bundle sheath chloroplasts increased with increasing water deficit to dissipate excess excitation energy. Interestingly, the NPQ in bundle sheath chloroplasts was higher than in mesophyll chloroplasts when the plants were well watered. Furthermore, with increasing drought stress, the NPQ in bundle sheath chloroplasts displayed a greater increase than in mesophyll chloroplasts. As shown in light response curves (LRCs) analysis, compared with mesophyll chloroplasts, the primary energy conversion of bundle sheath chloroplasts decreased more slightly, while the NPQ of bundle sheath chloroplasts increased more obviously.

**Figure 4:**
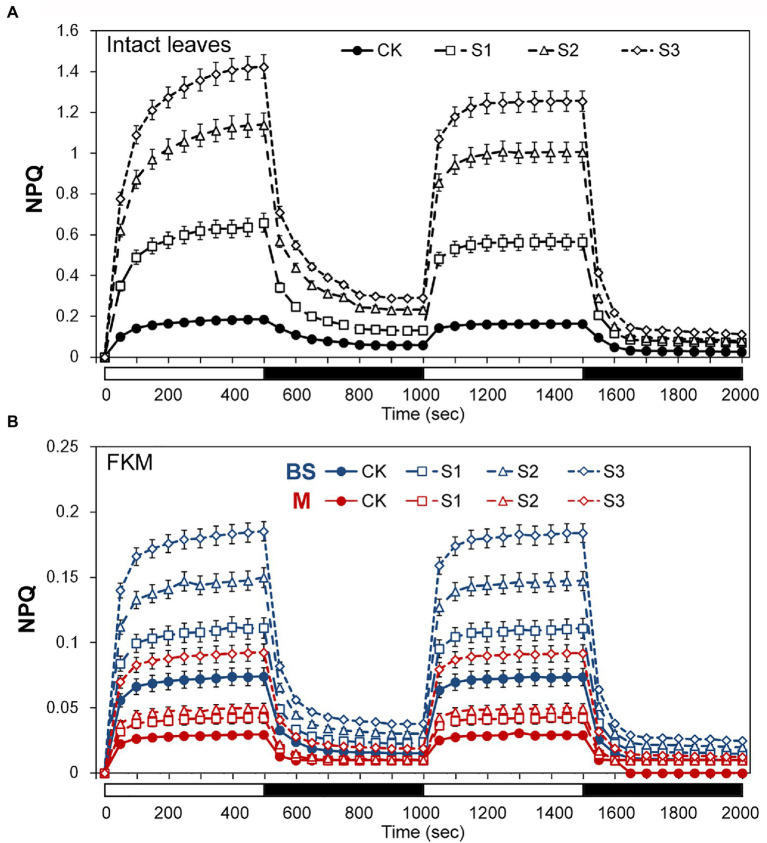
Assays of non-photochemical quenching (NPQ) kinetics under drought stress. **(A)** Measurement of NPQ kinetics in intact maize leaves. **(B)** Measurement of NPQ kinetics in M and BS chloroplasts of maize leaves. The two consecutive periods of illumination with 1,500 mol m^−2^ s^−1^ for 500 s with a 500 s period of darkness in between, as indicated by the white (light on) and black (dark) bars at the bottom of figure. CK, well-watered control; S1, mild drought stress; S2, moderate drought stress; and S3, severe drought stress. Vertical bars represent SD of the mean (*n* = 4).

The above results seemed to indicate that bundle sheath chloroplasts of maize leaves may be highly capable of thermal dissipation, especially under drought conditions. NPQ kinetic studies were performed in intact leaves and leaf transections with a light (1,500 mmol m^−2^ s^−1^) over a time period of 0–2,000 s. Under the high-light periods, drought treatment induced a typical rapid increase in NPQ followed by a slower increase, both in intact leaves and microstructure samples. The increasing trend of NPQ was positively correlated with the degree of drought stress (Figure 4). In particularly, as shown in Figure 4B, mesophyll chloroplasts exhibited a significantly weaker increase of NPQ than bundle sheath chloroplasts.

### Drought stress decreased phospho-lhcii level, especially in bundle sheath chloroplasts

The accumulation of PSII proteins in bundle sheath chloroplasts can be determined when cross-contamination with mesophyll cells is avoided. The mesophyll and bundle sheath chloroplasts of maize leaves exposed to drought stress were obtained through mechanical isolation. To determine the separation efficiency, isolated mesophyll and bundle sheath cell samples were immuno-blotted with the antibodies against mesophyll-specific enzyme PEPC and bundle sheath-specific enzyme Rubisco. The result is shown in Supplementary Figure 3A, almost no PEPC was detected in bundle sheath cells, and Rubisco was detected only in bundle sheath chloroplasts. Under drought stress, especially severe drought, the steady-state levels of PEPC and Rubisco declined. Thus, the used procedure effectively isolated mesophyll and bundle sheath cells, and was suitable for our investigation.

**Figure 5:**
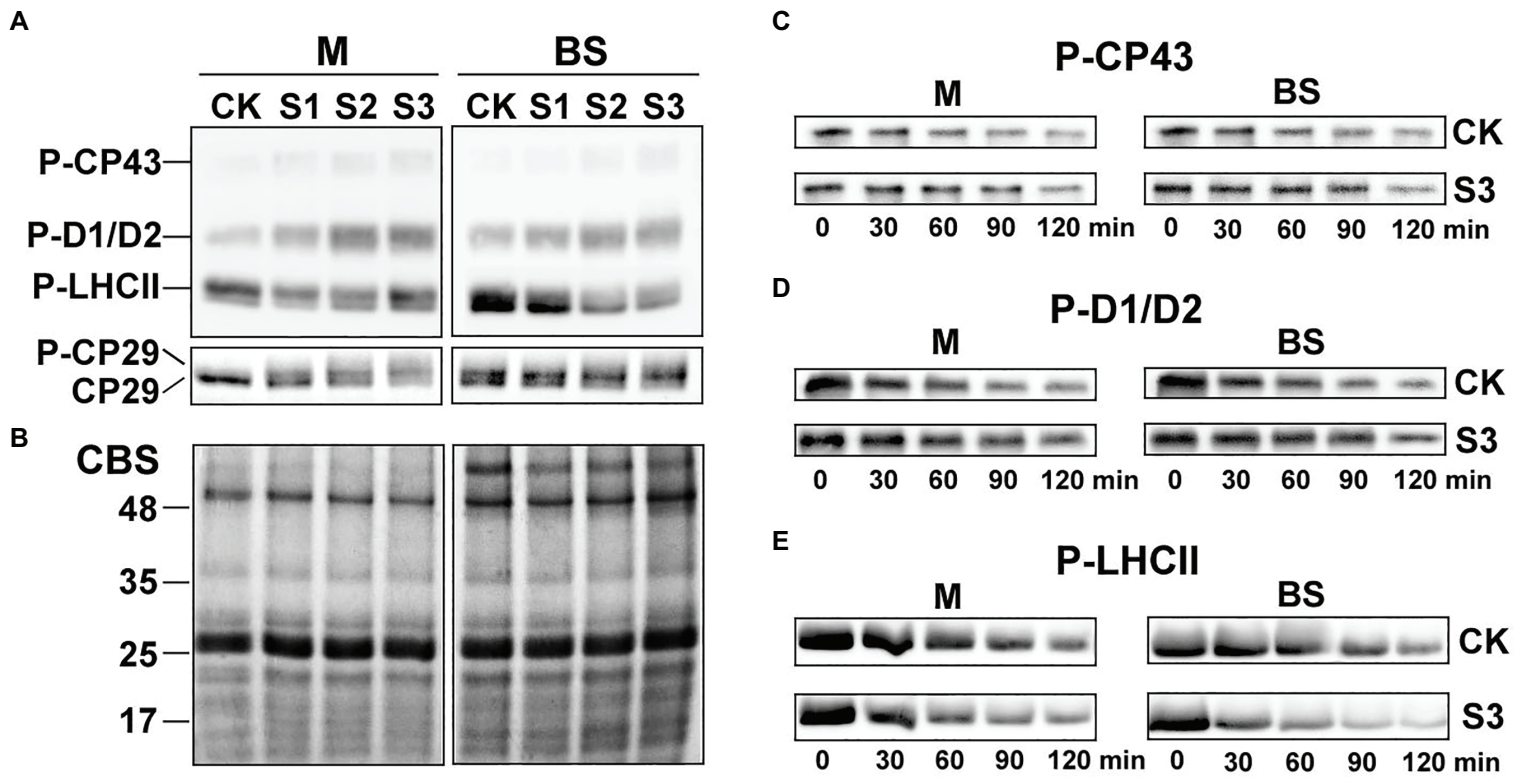
Reversible phosphorylation of PSII proteins in M and BS chloroplasts of maize leaves under drought stress. **(A)** Immunoblot analysis of the PSII proteins phosphorylation in M and BS chloroplasts under drought stress. Proteins in M (1.0 μg Chl) and BS chloroplasts (1.5 μg Chl), were detected with anti-PThr antibody. **(B)** Coomassie staining of protein samples (CBS) was shown as a control. The positions of detected phosphoproteins and molecular masses of protein markers (in kDa) are indicated. **(C–E)** Dephosphorylation of PSII proteins *in vivo* under severe drought stress. Maize leaves were illuminated 120 min at 25°C and then transferred to darkness and incubated at 25°C. Dephosphorylation was terminated at the indicated time points by freezing the leaves in liquid nitrogen. Thylakoid membranes in M and BS cells were isolated and the extent of protein phosphorylation was determined using anti-PThr antibody. Before conducting the dephosphorylation experiments, different light intensities were used for induction of higher *in vivo* phosphorylation levels of either core proteins or LHCII. Maize leaves were illuminated under a PFD 1,000 μmol photons m^−2^ s^−1^ for more effective phosphorylation of PSII core proteins **(C,D)** or under a PFD 80 μmol photons m^−2^ s^−1^ for induction of LHCII phosphorylation **(E)**. CK, well-watered control; S1, mild drought stress; S2, moderate drought stress; and S3, severe drought stress. The results shown are representative of those obtained in at least three independent experiments. Thylakoids were isolated in the presence of 10 mM NaF.

As shown in Figure 5A, the steady-state levels of phosphorylated CP43, D1, and D2 in the mesophyll and bundle sheath cells increased significantly under drought stress. On the contrary, the levels of phospho-LHCII decreased dramatically during drought treatment, especially in bundle sheath chloroplasts. The reversible phosphorylation of Lhcb4 (CP29) during water stress has been reported in our previous studies (Liu et al., 2009). In this experiment, phospho-CP29 was not detected in mesophyll chloroplasts but was detected in bundle sheath chloroplasts when the maize plants were well watered. Drought stress increased CP29 phosphorylation, in both mesophyll and bundle sheath chloroplasts.

The dephosphorylation rates of the core PSII proteins and LHCII were investigated in this study to better understand the dynamic changes in the phosphorylation status of the PSII proteins under drought stress. The dephosphorylation rates of the core PSII proteins (CP43, D1, and D2) in maize plants under severe drought stress were lower than in well-watered plants (Figures 5C,D). In contrast, drought treatment led to an obvious increase in the dephosphorylation rate of LHCII (Figure 5E). Under severe drought, the half-times of phospho-LHCII decreased from 74 and 94 min to 48 and 34 min in mesophyll and bundle sheath chloroplasts, respectively. This indicated that the dephosphorylation of LHCII proteins was more accelerated in bundle sheath chloroplasts than in mesophyll chloroplasts under drought treatment (Table 1).

**Table 1:** Dephosphorylation rates for CP43, D1/D2, and LHCII phosphoproteins in isolated mesophyll (M) and bundle sheath (BS) thylakoids under drought stress.

Phosphoprotein	*t*_1/2_ (min)							
	Mesophyll				Bundle sheath			
	CK	S1	S2	S3	CK	S1	S2	S3
CP43	96 ± 5^d^	112 ± 9^c^	132 ± 11^b^	164 ± 15^a^	64 ± 5^e^	88 ± 7^d^	103 ± 9^c^	139 ± 13^b^
D1/D2	76 ± 6^d^	93 ± 8^c^	111 ± 10^b^	143 ± 16^a^	52 ± 3^e^	75 ± 7^d^	98 ± 10^bc^	131 ± 12^a^
LHCII	74 ± 4^b^	66 ± 5^bc^	57 ± 4^c^	48 ± 4^d^	94 ± 8^a^	78 ± 7^b^	55 ± 5^c^	34 ± 2^e^

The data are presented as the half-times (minutes). The half-times were calculated from the first-order rate fitting of the dephosphorylation versus time curves obtained from four experiments at each treatment. CK, well-watered control; S1, mild drought stress; S2, moderate drought stress; and S3, severe drought stress. Results are expressed as means ± SD of four independent experiments, different letters mean significant differences at the 0.05 level according to Duncan’s multiplication range test.

### The changes in steady-state levels of psii proteins differed between mesophyll and bundle sheath chloroplasts in response to drought stress

**Figure 6:**
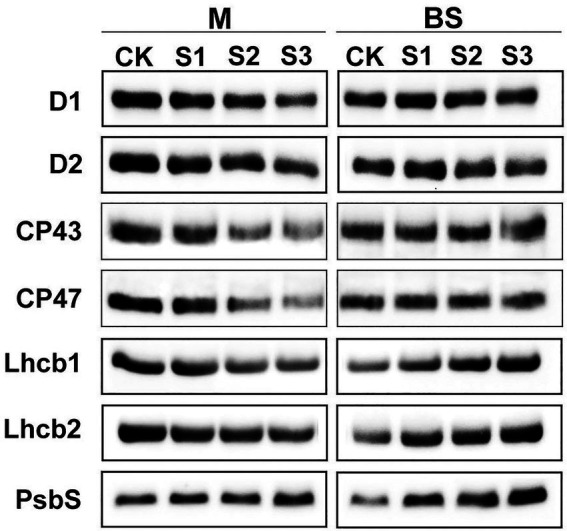
Steady-state levels of PSII proteins in M and BS thylakoids of maize leaves under drought stress. Immunoblot analyses of PSII proteins in M thylakoids (1.0 μg Chl) and BS thylakoids (1.5 μg Chl) were performed using antibodies specific for D1, D2, CP43, CP47, Lhcb1, Lhcb2, and PsbS. The results shown are representative of those obtained in at least three independent experiments. CK, S1, S2, and S3 represent, respectively, the well-watered, mild drought, moderate drought and severe drought treatments.

The steady-state levels of PSII proteins in mesophyll and bundle sheath chloroplasts of maize leaves subjected to drought stress were investigated by immunoblot analysis (Figure 6). In general, PSII protein levels in bundle sheath were much lower than those in mesophyll cells, which is consistent with the previous reports (Majeran et al., 2005, 2008). In mesophyll chloroplasts, the levels of the core PSII proteins were all markedly reduced under drought stress (Supplementary Figures 4A–F). Similar trends were not observed in bundle sheath chloroplasts under drought conditions. Interestingly, the LHCII content in bundle sheath chloroplasts increased when plants were subjected to drought stress.

It is considered that the PSII protein, PsbS, could play a crucial role in enabling the rapidly reversible component of NPQ (Li et al., 2000). Therefore, the response of the PsbS level upon drought stress was investigated in this experiment. As shown in Figure 6, the steady-state levels of PsbS in both mesophyll and bundle sheath chloroplasts increased under drought stress. Furthermore, this increase was greater in bundle sheath chloroplasts than in mesophyll chloroplasts. After severe drought, the levels of PsbS in the mesophyll and bundle sheath chloroplasts increased by 28.20% and 124.87%, respectively, compared with the control (Supplementary Figure 4G).

### The organization of psii complexes in mesophyll and bundle sheath thylakoids varied under drought stress

PSII is present in thylakoid membranes both as a dimer and a monomer. The functional dimeric PSII complexes bind at least two LHCII trimers, thus forming the PSII-LHCII supercomplexes in the appressed grana regions. Some evidence suggests that, during NPQ, PsbS controls the dissociation of the portion of PSII-LHCII supercomplexes and aggregation of LHCII antenna (Betterle et al., 2009; Johnson and Ruban, 2011; Goral et al., 2012). In our study, the aim was to gain an insight into the organizational changes of the PSII complexes in thylakoid membranes in the leaves of maize plants subjected to drought stress. To do this, the thylakoid membranes in mesophyll and bundle sheath chloroplasts were solubilized with 1% and 2% DM, respectively. The thylakoids were then analyzed using the blue native PAGE (BN-PAGE) technique.

**Figure 7:**
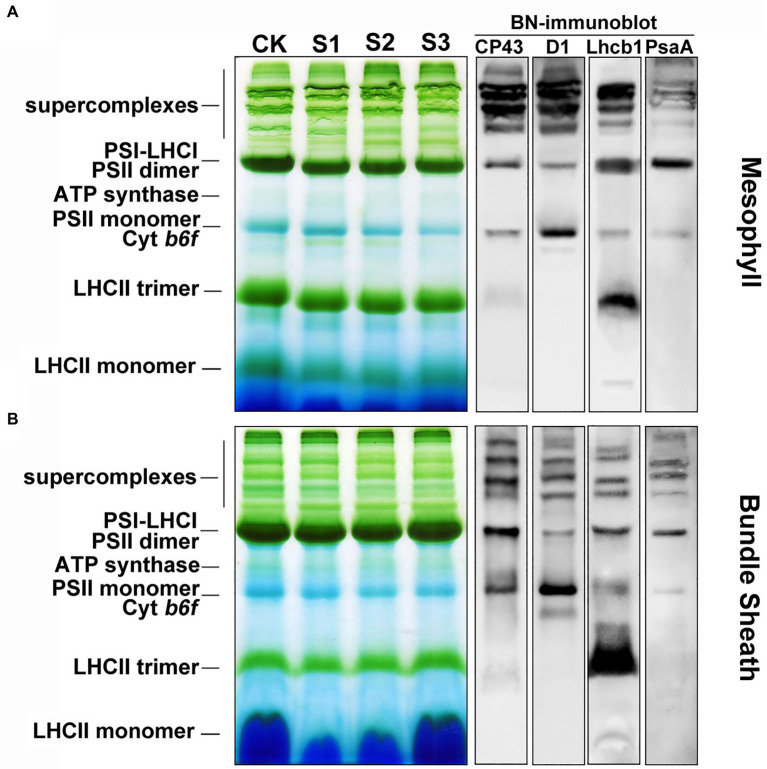
The composition of protein complexes in M and BS thylakoids of maize leaves under drought stress. **(A)** Membranes (15 μg Chl) in M thylakoids were solubilized with 1% n-dodecyl β-D-maltoside (DDM) and loaded onto 4%–12% acrylamide Blue-Native gel. **(B)** Membranes (15 μg Chl) in BS thylakoids were solubilized with 2% DDM and loaded onto 4%–12% acrylamide Blue-Native gel. The bands of BN-PAGE were confirmed by immunoblotting with CP43, D1, Lhcb1, and PsaA specific antibodies (on the right). The control line of the BN gel was selected for immunodetection. The results shown are representative of those obtained in at least three independent experiments. CK, S1, S2, and S3 represent, respectively, the well-watered, mild drought, moderate drought and severe drought treatments.

Protein analysis of crosslinked thylakoids using BN-PAGE revealed that the composition of protein complexes in mesophyll and bundle sheath thylakoids were similar to those described by Pokorska et al. (2009) and Rogowski et al. (2018). Bands corresponding to major protein complexes were identified on BN-gels (Figure 7). These complexes included the PSII-LHCII and PSI-LHCI supercomplexes, PSII dimers and monomers, ATP synthase, PSI core complex, and LHCII trimers. The relative level of the supercomplexes was lower in bundle sheath thylakoids compared with mesophyll thylakoids, and the abundance of the LHCII trimers was lower in bundle sheath membranes. As expected, drought stress induced different changes to the steady-state levels of protein complexes in mesophyll and bundle sheath thylakoids. There were reductions in the amount of PSII dimers, PSII monomers, and LHCII trimers in mesophyll thylakoids isolated from maize leaves under drought stress when compared to the well-watered control (Figure 7A). These reductions were greater in thylakoids isolated from maize subjected to severe drought stress. Nevertheless, no such changes were observed in bundle sheath membranes. When maize leaves were exposed to drought stress, almost all the protein complexes in the bundle sheath thylakoids were stable (Figure 7B). Interestingly, severe drought stress markedly increased the relative abundance of LHCII trimers in bundle sheath membranes (Supplementary Figure 5).

### Drought stress accelerated the destacking of grana in mesophyll thylakoids

**Figure 8:**
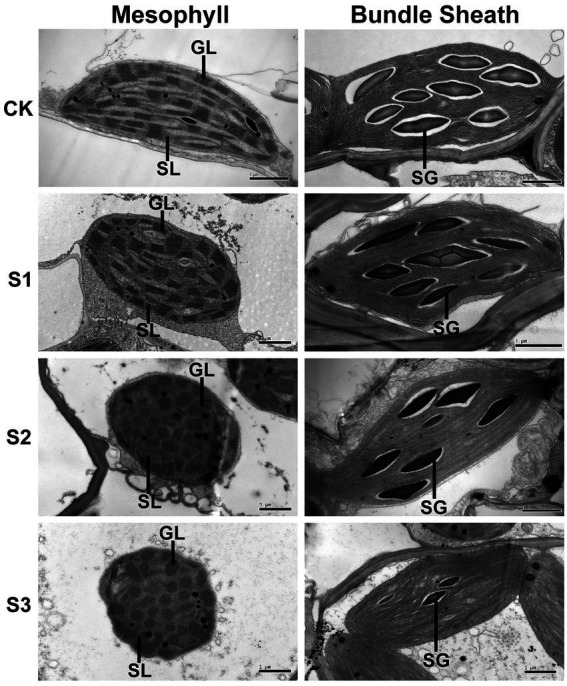
Transmission electron microscope analysis of chloroplasts in maize leaves exposed to progressive drought stress. GL and SL represent, respectively, the grana lamellae and stroma lamellae of mesophyll thylakoids, and SG represents starch grain. CK, well-watered control; S1, mild drought stress; S2, moderate drought stress; and S3, severe drought stress.

To further investigate the effects of drought stress on thylakoid structures, the ultrastructure of mesophyll and bundle sheath chloroplasts in maize leaves were analyzed (Figure 8). Transmission electron microscopy showed that the length-to-width ratio and area of mesophyll chloroplasts tended to decrease in response to drought stress. The stacking of the grana in mesophyll chloroplasts gradually loosened as drought stress increased. The number of grana also reduced. The bundle sheath chloroplasts appeared to lack grana, which is in accordance with the conclusion reported by Edwards et al. (2001). As shown in Figure 8, the unstacked thylakoid lamellae in bundle sheath chloroplasts were not obviously damaged after drought treatment. However, the starch granules were reduced both in size and in number under severe drought stress.

## Discussion

There are many notable photosynthetic structures and functions that differ between C4 and C3 plants. These include the Kranz anatomy, the higher optimum temperature and irradiance saturation for maximum photosynthetic rates, the lower CO_2_ compensation point, and the higher instantaneous water use efficiency of leaves of C4 plants (El-Sharkawy, 2009). These characteristics may provide C4 plants with a superior capacity for environmental adaptation. In our previous work, water deficit reduced stomatal opening in wheat and barley leaves to reduce water transpiration. This directly affected CO_2_ uptake and led to reduced CO_2_ assimilation and a corresponding decrease in energy conversion in PSII, even under mild water stress. Furthermore, excess excitation energy led to the production of ROS (including O_2_^−^, ^1^O_2_, H_2_O_2_, and OH). ROS accumulation has been shown to impair cell structure and damage photosynthetic apparatus, which further reduces the net photosynthetic rate and plant growth (Yuan et al., 2005; Liu et al., 2006, 2009). Nevertheless, in this study, reduced stomatal conductance in maize leaves only slightly affected the net photosynthetic rate under mild drought stress (Supplementary Figure 1). Significant increases in *C_i_* (Supplementary Figure 1E) and reductions in net photosynthetic rate (Supplementary Figure 1F) only occurred in leaves subjected to severe drought conditions. These results suggest that maize may have a mechanism for maintaining photosynthetic assimilation efficiency, thereby limiting the negative feedback of stomatal and non-stomatal influences under drought stress.

The discovery of functional PSII complexes in bundle sheath chloroplasts of maize provided a new insight into the photosynthetic mechanism of C4 plants (Romanowska et al., 2006; Pokorska et al., 2009; Rogowski et al., 2018). In this study, the presence of an NPQ mechanism in the bundle sheath chloroplasts of maize was determined through physiological and biochemical experiments. Importantly, more significant enhancement of NPQ was detected in bundle sheath chloroplasts under drought stress than in mesophyll chloroplasts (Figures 3, 4). NPQ is considered as the most rapid molecular response of PSII in higher plants for protecting photosynthetic apparatus. However, many aspects of the NPQ mechanism such as the quenching site and its regulation are still debated currently. Ruban (2016) summarized that the minimum requirements for NPQ *in vivo* are the proton gradient (ΔpH), LHCII complexes, and the PsbS protein. When light intensities change, ΔpH, as the trigger, results in PsbS being protonated. This leads to concomitant rearrangements of the antenna system, which switches the antenna into their dissipative state. In this quenched state, LHCII is dephosphorylated and dissociates from PSII-LHCII complexes. This favors thermal dissipation of excitation energy over energy transfer to RCII (Tikkanen and Aro, 2012). In the above model, PsbS protein functions as a switch. The PSII-LHCII pool and aggregated LHCII antenna participate in the energy-dependent quenching (qE; Ruban, 2016; Rogowski et al., 2018), in which dephosphorylation of LHCII may occur (Rintamäki et al., 1997).

PsbS content can be adjusted to the intensity of the growth light conditions (Ballottari et al., 2007). When grown in high light, plants have a faster induction and relaxation rate for qE, which is correlated with a higher abundance of the PsbS protein (Kromdijk et al., 2016). Our previous research has also shown that NPQ and PsbS content in Arabidopsis leaves increased under long-term (6–15 days) drought stress (Chen et al., 2016). In this study on maize, increasing drought stress led to an increase in NPQ (Figures 2–4), which was accompanied by accelerated dephosphorylation of the LHCII subunits (Figure 5) and enhanced PsbS content (Figure 6). These results were consistent with previous findings in C3 plants and were observed in both mesophyll and bundle sheath chloroplasts. Therefore, it can be concluded that LHCII antenna and the PsbS protein may play roles in regulating excess energy dissipation in PSII in maize leaves. The accumulation of PsbS may be positively correlated with the NPQ capacity when maize plants encounter drought stress.

More significantly, it can be deduced that the photoprotection capacity (*via* thermal dissipation) of bundle sheath chloroplasts is superior to that of mesophyll chloroplasts in response to drought stress. This may be significantly correlated with the dephosphorylation of LHCII (Table 1) and the PsbS levels (Supplementary Figure 4G) in bundle sheath chloroplasts. This superior capacity of bundle sheath cells was also reflected by the NPQ displayed in the two types of chloroplasts under drought stress. Additionally, the phosphorylation of core proteins (CP43, D1, and D2) and CP29 in both mesophyll and bundle sheath chloroplasts increased under drought stress. Interestingly, phospho-CP29 was found in bundle sheath chloroplasts but not mesophyll chloroplasts, when maize plants were well watered (Figure 5A). The phosphorylation of core proteins has been shown to have a role in facilitating the disassembly and migration of the PSII-LHCII supercomplexes under changing light intensities (Goral et al., 2010; Tikkanen and Aro, 2012; Chen et al., 2018a). The significance of the phosphorylation of CP29 is still controversial. Some researchers have suggested that the phosphorylation of CP29 is associated with NPQ (Betterle et al., 2015). It has even been proposed that the quencher is localized within CP29 (Ahn et al., 2008). Whether the NPQ mechanism in mesophyll and bundle sheath chloroplasts of maize under drought stress involves the reversible phosphorylation of CP29 requires further study.

Previous studies have shown that bundle sheath cells exhibit predominantly PSI cyclic electron flow, which generates a trans-thylakoid ΔpH (Ivanov et al., 2005, 2007). This feature of bundle sheath cells may be also correlated with the higher NPQ bundle sheath cells and may be not attributed into a greater need for photoprotection under drought. The roles of higher PSI cyclic electron flow in bundle sheath cells under environmental stresses require further investigations.

Inspection of the histochemical staining of O_2_^−^ and H_2_O_2_ indicated that drought stress resulted in higher ROS accumulation in mesophyll cells than in bundle sheath cells (Figures 1A,B). The results of measuring the plastid MDA also showed that the lipid peroxidation of chloroplast membranes in mesophyll cells was more severe than in bundle sheath cells under drought conditions (Figure 1C). The homeostasis between the formation and removal of ROS in plant cells is regulated through enzymatic pathways and antioxidants. Increased ROS accumulation under environmental stress can damage proteins, membrane lipids, DNA, and other cellular components. NPQ, as the first defense and photoprotection mechanism of PSII, exerts control over the CO_2_ assimilation rate in fluctuating light conditions (Hubbart et al., 2012; Roach and Krieger-Liszkay, 2012; Zulfugarov et al., 2014).

Consistent with our previous findings in wheat, barley, and Arabidopsis (Liu et al., 2006, 2009; Chen et al., 2016), ROS accumulation may be one of the main reasons for the increase in lipid peroxidation (Figure 1C), downregulation of photosynthetic proteins (Figures 6, 7), and destacking of grana (Figure 8) in mesophyll chloroplasts of maize under drought stress. In bundle sheath cells, after drought treatment, there was no obvious peroxidative damage or reductions in protein abundance. Interestingly, the steady-state levels of LHCII (including the levels of the LHCII monomer and the LHCII trimer) in the bundle sheath chloroplasts increased with increasing drought stress (Figures 6, 7B). This may have contributed to maintaining the higher NPQ, as LHCII may be important to the NPQ mechanism (Rintamäki et al., 1997; Ilioaia et al., 2011). Therefore, our data indicate that the lower accumulation of ROS in bundle sheath chloroplasts under drought conditions compared to that in the mesophyll chloroplasts may be related to the superior capacity of bundle sheath cells to dissipate excess energy.

Maize, as an NADP-dependent malic enzyme (NADP-ME) type of C4 plants, has a CO_2_ concentrating mechanism in its bundle sheath cells coupled with high stomatal resistances. This results in improved water conservation in leaves when the CO_2_ assimilation efficiency is equal to or higher than that of C3 plants (El-Sharkawy, 2009). Therefore, when environmental factors change, it is crucial for maize to maintain photosynthetic efficiency by ensuring the CO_2_ assimilation capacity in bundle sheath chloroplasts and limiting CO_2_ diffusion from bundle sheath cells to mesophyll cells (Langdale, 2011). The superior NPQ mechanism in bundle sheath cells inhibits the release of ROS to an extent, which may have positive consequences for maintaining the structure and function of bundle sheath cells. This enables the bundle sheath cells to retain high CO_2_ concentrations and improves the water use efficiency of leaves under drought conditions. In addition, the lower accumulation of ROS under drought stress could reduce the risk of oxidative damage to the transitory starch stored in bundle sheath chloroplasts. This starch can be utilized as a stored energy source for glucose metabolism in maize cells (Figure 8). Currently, there are no suitable instruments or methods to separately measure the water potential or osmotic pressure in mesophyll and bundle sheath cells of plants with dense veins such as maize. Here, we presume that the structural integrity and functional stability of bundle sheath cells under drought stress may contribute to the ability of vascular tissues to transport water, mineral salts, and organic compounds produced by photosynthesis.

Many studies on photosynthesis in C4 plants focus on the CO_2_ assimilation process; researchers attempt to improve the photosynthetic efficiency, especially under environmental stress, through modifications such as the enhancement of PEPC content and activity. There are many strategies for improving the photosynthetic capacity of C3 plants that have been put into practice. These include the introduction of Rubisco or PEPC from C4 species, the introduction of carbon concentrating mechanisms, and the engineering of a full C4 Kranz pathway using the existing evolutionary progression observed in C3–C4 intermediates as a blueprint (Leegood, 2013; Qin et al., 2015).

The effect of PsbS expression on NPQ has been well-documented (Peterson and Havir, 2001, 2003). C3 plants such as Arabidopsis, tobacco, and rice overexpressing PsbS have been shown to display increased photoprotection compared with wild-type plants under high light or other fluctuating environmental conditions (Roach and Krieger-Liszkay, 2012; Glowacka et al., 2018). However, this can be at the expense of CO_2_ fixation under less stressful conditions (Hubbart et al., 2012; Kromdijk et al., 2016). In this study in maize, the significant correlation between PsbS content and NPQ was found in both mesophyll and bundle sheath chloroplasts. The superior photoprotection observed in bundle sheath cells may be beneficial to stabilizing their function. Therefore, it is suggested that overexpression of PsbS in C4 plants such as maize may improve environmental adaptation. Moreover, the PsbS protein, particularly in bundle sheath chloroplasts, is hypothesized to be a useful marker for assessing chlorophyll fluorescence and field yield when breeding stress-resistant maize varieties. This hypothesis has recently been discussed in the context of wheat (Chen et al., 2018b).

## Data availability statement

The original contributions presented in the study are included in the article/Supplementary Material; further inquiries can be directed to the corresponding authors.

## Author contributions

W-JL, SY, and JZ planned and designed the research and wrote the manuscript with contribution of all authors. W-JL, HL, Y-EC, YY, Z-WZ, JS, L-JC, F-LZ, and DW performed experiments. W-JL, HL, X-HD, CW, MX, SY, and JZ analyzed data. All authors contributed to the article and approved the submitted version.

## Funding

This work was supported by Beijing Municipal Joint Research Program for Germplasm Innovation and New Variety Breeding (to JZ), the National Natural Science Foundation of China (31770322 to SY), the National Special Program for GMO Development of China (2016ZX08003-004 to JZ), the Natural Science Foundation of Sichuan Province (2022NSFSC0140 to W-JL), the Fund of Talent Project of Sichuan Academy of Agricultural Sciences (2021LJRC to W-JL), and the Fund of “1+9” Science and Technology Project of Sichuan Academy of Agricultural Sciences (1+9 KJGG 006), Advanced technology for biosafety.

## Conflict of interest

The authors declare that the research was conducted in the absence of any commercial or financial relationships that could be construed as a potential conflict of interest.

## Publisher’s note

All claims expressed in this article are solely those of the authors and do not necessarily represent those of their affiliated organizations, or those of the publisher, the editors and the reviewers. Any product that may be evaluated in this article, or claim that may be made by its manufacturer, is not guaranteed or endorsed by the publisher.

## Supplementary materials

The Supplementary materials for this article can be found online at: https://www.frontiersin.org/articles/10.3389/fpls.2022.885781/full#supplementary-material
