# Supplementary material for: Chloroplastic photoprotective strategies differ between bundle sheath and mesophyll cells in maize (Zea mays L.) Under drought
Source: Front Plant Sci. 2022 Jul 14;13:885781. doi: 10.3389/fpls.2022.885781 (PMC9330506; doi:10.3389/fpls.2022.885781)
Supplement: Supplementary file 1 [file Data_Sheet_1.PDF]

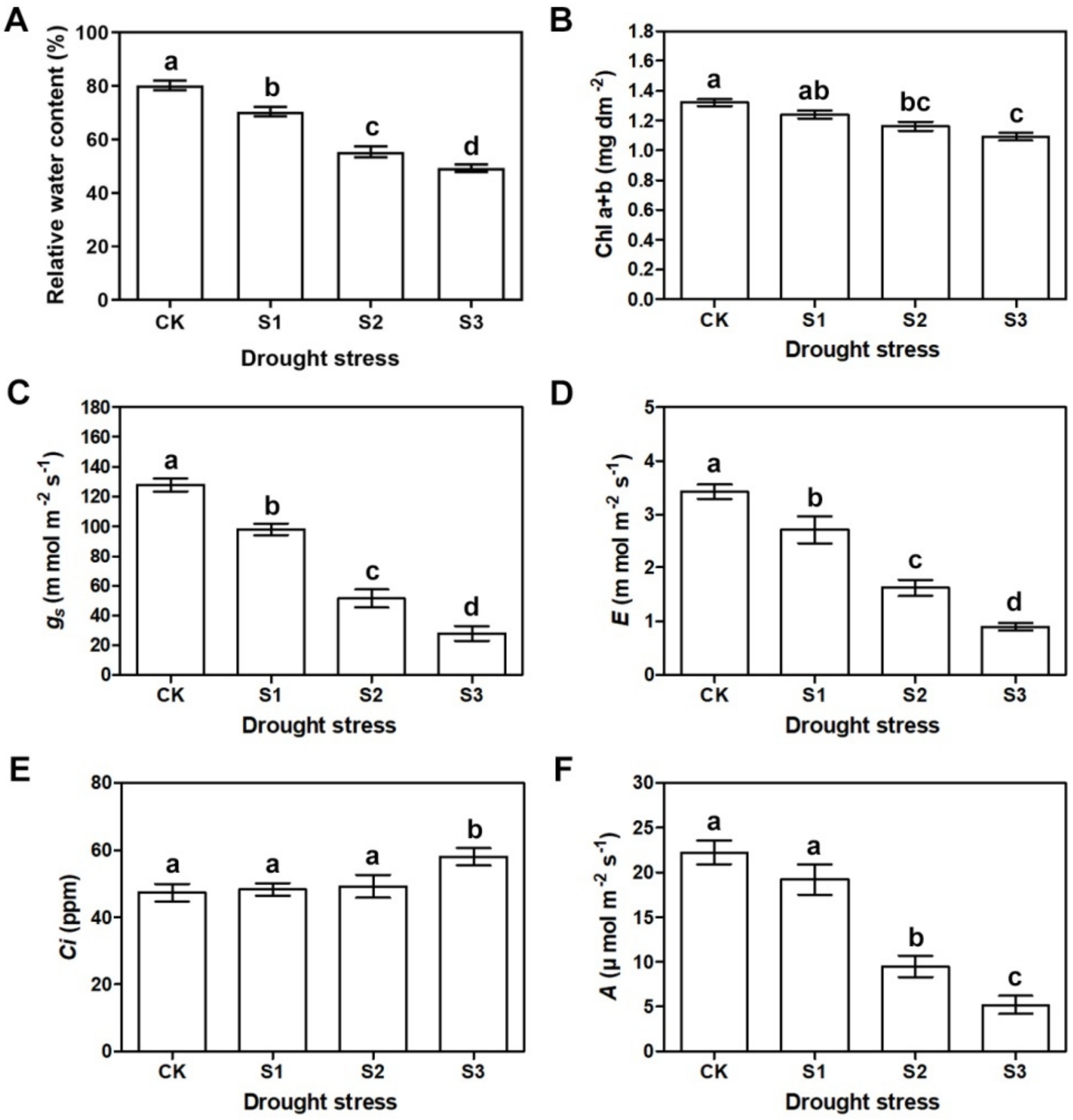

SUPPLEMENTARY FIGURE1 | Measurement of RWC **(A)**, contents of Chl a and b **(B)**, and gas exchange in maize leaves under drought stress. Stomatal conductance g_s **(C)**, transpiration rate *E* **(D)**, intercellular CO_2 concentration C_i **(E)** and net photosynthetic rate *A* **(F)** in leaves were measured to reflect the influence of drought stress on gas exchange. CK, well watered control; S1, mild drought stress; S2, moderate drought stress; S3, severe drought stress. Each point represents the mean±SD (n=4). Different letters mean significant differences at the 0.05 level according to Duncan's multiplication range test.

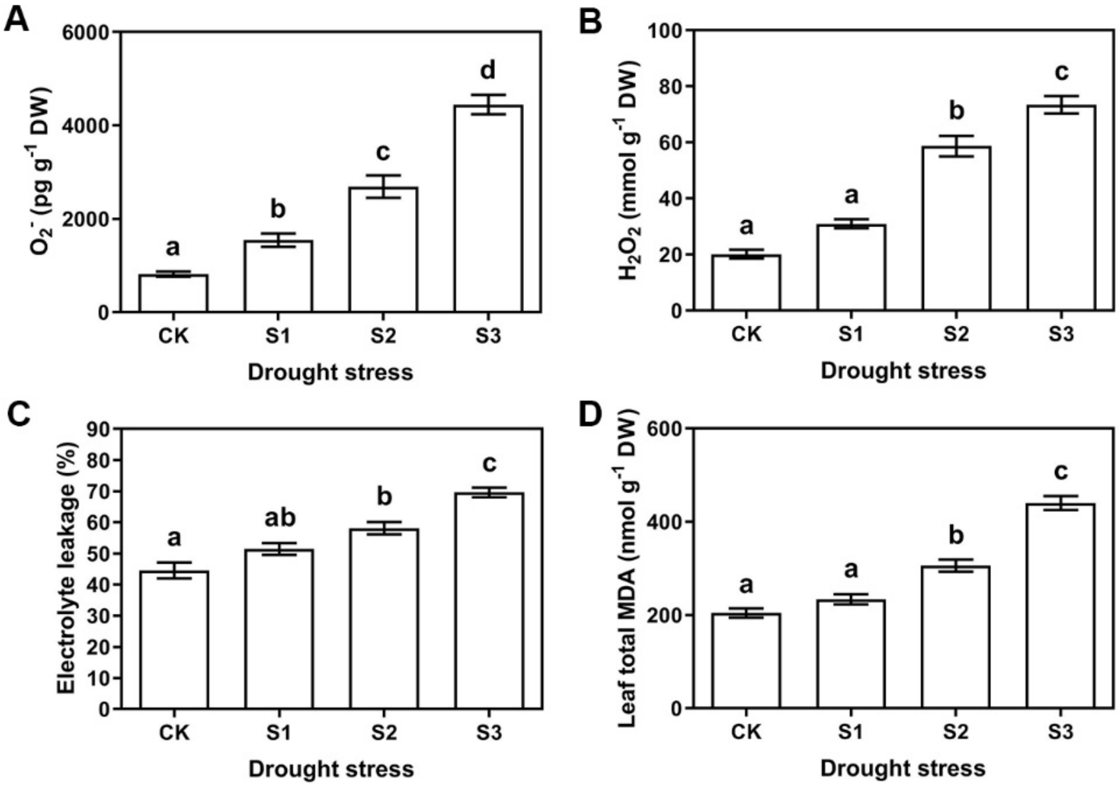

SUPPLEMENTARY FIGURE 2 | Measurement of ROS and lipid peroxidation in maize leaves under drought stress. Superoxide anion radicals (O_2^-) **(A)** and H_2O_2 **(B)** production were measured to determine the ROS level. Electrolyte leakage **(C)** and leaf total MDA content **(D)** were examined to estimate the lipid peroxidation of maize leaves. CK, S1, S2 and S3 represent respectively the soil moisture regimes of well watered, mild drought stress, moderate drought stress and severe drought stress. Vertical bars represent SD of the mean (*n*=4). Different letters mean significant differences at the 0.05 level according to Duncan's multiplication range test.

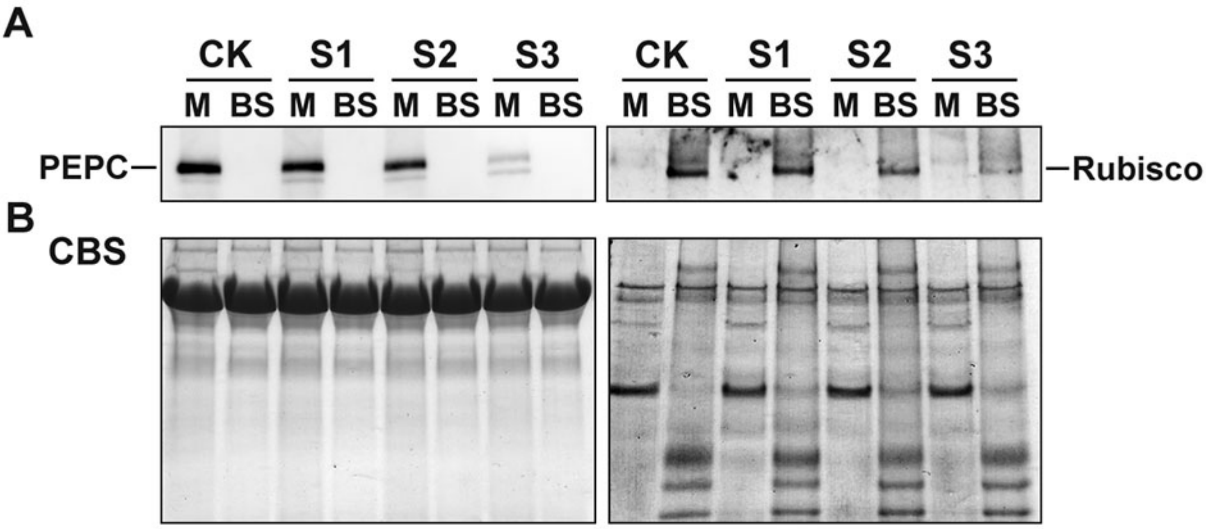

SUPPLEMENTARY FIGURE 3 | Analysis of effective isolation of mesophyll and bundle sheath cells (mechanical isolation) from maize leaves. **(A)** Immunoblot analysis of PEPC and Rubisco proteins in mesophyll (M) and bundle sheath (BS) cells under drought stress. PEPC in M and BS soluble fractions (2.0 μg proteins) were detected with anti-PEPC antibody, and Rubisco in M and BS chloroplasts (0.5 μg Chl) were detected with anti-Rubisco antibody. **(B)** Coomassie staining of protein samples (CBS) in soluble fractions and chloroplastswere shown as a control. CK, well watered control; S1, mild drought stress; S2, moderate drought stress; S3, severe drought stress.

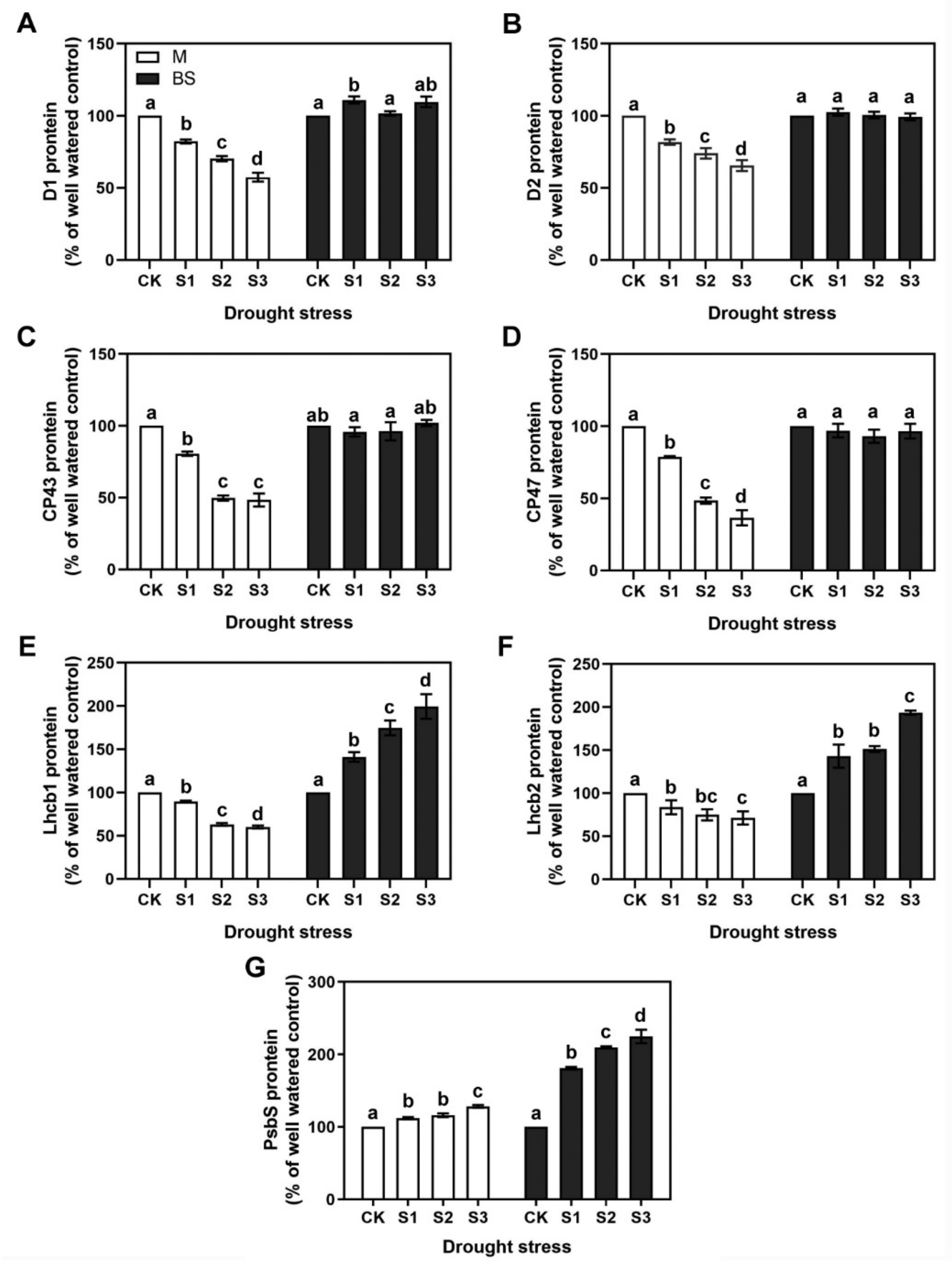

SUPPLEMENTARY FIGURE 4 | Quantification of PSII protein immunoblot signals in mesophyll (M) and bundle sheath (BS) thylakoids isolated from maize leaves under drought stress. CK, S1, S2 and S3 represent respectively the soil moisture regimes of well watered, mild drought stress, moderate drought stress and severe drought stress. The amounts of proteins under well watered condition were defined as 100%. Results are expressed as means ± SD of four independent experiment, different letters mean significant differences at the 0.05 level according to Duncan's multiplication range test.

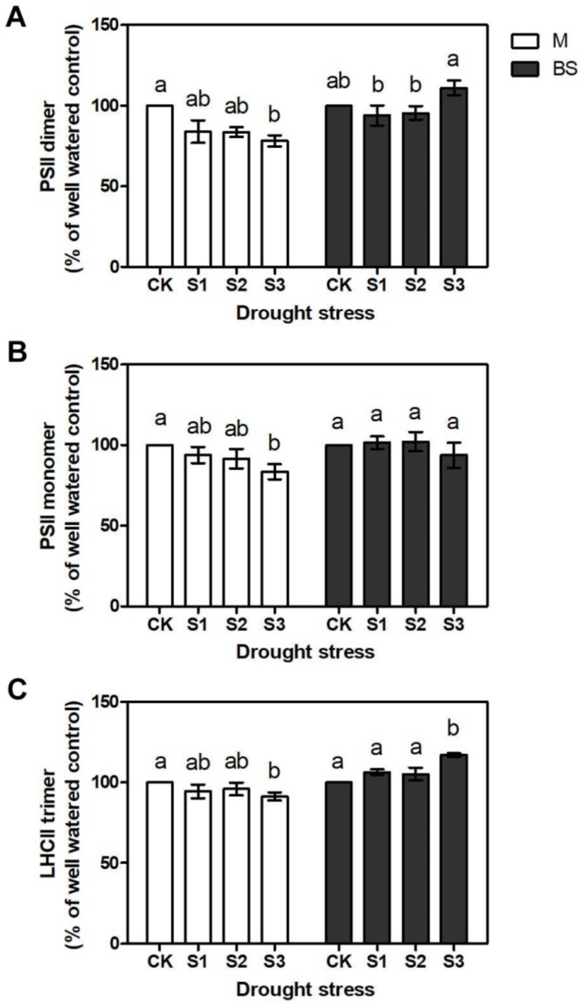

SUPPLEMENTARY FIGURE 5 | Quantification of the primary PSII complexes in mesophyll (M) and bundle sheath (BS) thylakoids isolated from maize leaves under drought stress. The levels of the complexes under well watered condition were defined as 100%. Vertical bars represent SD of the mean (*n*=4). Different letters mean significant differences at the 0.05 level according to Duncan's multiplication range test.
